# Phenylethyl isothiocyanate reverses cisplatin resistance in biliary tract cancer cells via glutathionylation-dependent degradation of Mcl-1

**DOI:** 10.18632/oncotarget.7171

**Published:** 2016-02-03

**Authors:** Qiwei Li, Ming Zhan, Wei Chen, Benpeng Zhao, Kai Yang, Jie Yang, Jing Yi, Qihong Huang, Man Mohan, Zhaoyuan Hou, Jian Wang

**Affiliations:** ^1^ Department of Biliary-Pancreatic Surgery, Ren Ji Hospital, School of Medicine, Shanghai Jiao Tong University, Shanghai, China; ^2^ Department of Biochemistry and Molecular Cell Biology, Shanghai Key Laboratory of Tumor Microenvironment and Inflammation, Institutes of Medical Sciences, Shanghai Jiao Tong University, School of Medicine, Shanghai, China; ^3^ The Wistar Institute, University of Pennsylvania and Veterans Affairs Medical Center, Philadelphia, Pennsylvania, USA

**Keywords:** biliary tract cancer, PEITC, cisplatin, Mcl-1, glutathionylation

## Abstract

Biliary tract cancer (BTC) is a highly malignant cancer. BTC exhibits a low response rate to cisplatin (CDDP) treatment, and therefore, an understanding of the mechanism of CDDP resistance is urgently needed. Here, we show that BTC cells develop CDDP resistance due, in part, to upregulation of myeloid cell leukemia 1 (Mcl-1). Phenylethyl isothiocyanate (PEITC), a natural compound found in watercress, could enhance the efficacy of CDDP by degrading Mcl-1. PEITC-CDDP co-treatment also increased the rate of apoptosis of cancer stem-like side population (SP) cells and inhibited xenograft tumor growth without obvious toxic effects. *In vitro*, PEITC decreased reduced glutathione (GSH), which resulted in decreased GSH/oxidized glutathione (GSSG) ratio and increased glutathionylation of Mcl-1, leading to rapid proteasomal degradation of Mcl-1. Furthermore, we identified Cys16 and Cys286 as Mcl-1 glutathionylation sites, and mutating them resulted in PEITC-mediated degradation resistant Mcl-1 protein. In conclusion, we demonstrate for the first time that CDDP resistance is partially associated with Mcl-1 in BTC cells and we identify a novel mechanism that PEITC can enhance CDDP-induced apoptosis via glutathionylation-dependent degradation of Mcl-1. Hence, our results provide support that dietary intake of watercress may help reverse CDDP resistance in BTC patients.

## INTRODUCTION

Biliary tract cancer (BTC) refers to a group of cancers of the biliary tract, including gallbladder cancer, cholangiocarcinoma of intrahepatic and extrahepatic bile ducts, and cancers of the ampulla and papilla of Vater [1]. BTC is a common form of cancer in East Asia and Latin America [2, 3] and surgical resection is the only curative treatment. However, most patients are diagnosed with advanced-stage disease, making them ineligible for complete surgical resection. The prognosis for patients with advanced BTC is very poor, and most survive for less than a year after diagnosis. Cisplatin (CDDP) based chemotherapy is widely used to treat patients with advanced BTC [4]. However, BTC cells are highly chemoresistant. The mechanism of CDDP resistance in BTC cells is poorly understood. Therefore, investigation into the mechanism of CDDP resistance and strategies to alleviate the CDDP resistance are urgently needed.

Various naturally occurring compounds are being tested for their anti-tumor activity. Such compounds when used in combination with known chemotherapeutic agents may help in overcoming chemoresistance, and may provide new strategies and ideas for treatment in clinic. Phenylethyl isothiocyanate (PEITC) is present in high concentrations as its precursor gluconasturtiin in cruciferous vegetables, such as watercress. Upon chewing or chopping, PEITC is released as a product of hydrolysis mediated by myrosinase [5]. Accumulating evidence indicates that PEITC can inhibit cell growth and induce apoptosis in a variety of cancer cells, suggesting its potential value as an anticancer agent or an adjunctive therapy to current cancer treatment strategies [6–8]. Additionally, it was shown that eating watercress can significantly increase the blood level of PEITC in humans [9]. These studies and their results gave us important clue to study the effect of combined treatment with PEITC and CDDP on BTC cells.

Though recent data has shown that PEITC can sensitize some tumor cells to CDDP [5, 10–12], the synergistic effect of PEITC and CDDP in BTC cells has not been investigated. Here, we demonstrate for the first time that CDDP resistance is partially associated with Mcl-1 in BTC cells, and PEITC can enhance CDDP-induced apoptosis via glutathionylation-dependent degradation of myeloid cell leukemia 1 (Mcl-1). Interestingly and promisingly, PEITC-CDDP co-treatment can increase the rate of apoptosis of cancer stem-like side population (SP) cells and significantly reduce the growth of xenograft tumor without any major toxic effects. Our results suggests that dietary intake of watercress, which is a rich source of PEITC, may help reverse CDDP resistance in BTC patients.

## RESULTS

### PEITC enhances CDDP-induced inhibition of cell viability in BTC cells by increasing apoptosis

**Figure 1:**
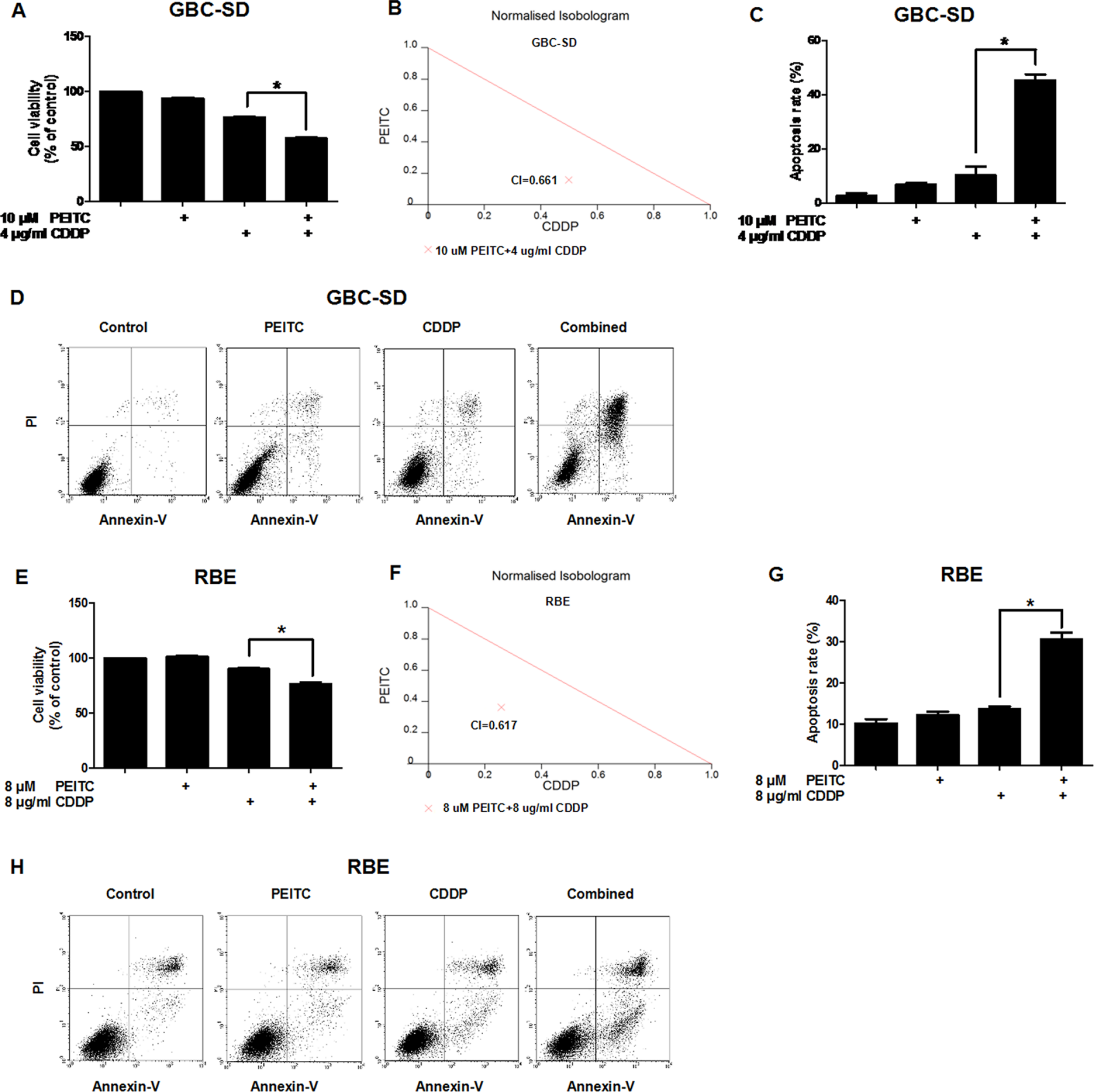
PEITC enhances CDDP-induced apoptosis in GBC-SD and RBE cells (**A**) Cells were treated with PEITC, CDDP or PEITC-CDDP combination for 24 hrs and prepared for MTT assays. (**B**) CI of PEITC-CDDP treatment in GBC-SD cells. (**C**) Flow cytometry analysis of rate of apoptosis in GBC-SD cells (Annexin V/PI flow cytometry, bar charts). (**D**) Apoptosis in GBC-SD cells (density plots). (**E**) Cell viability assay of RBE cells (MTT). (**F**) CI in RBE cells. (**G**) Flow cytometry analysis of rate of apoptosis in RBE cells (Annexin V/PI flow cytometry, bar charts). (**H**) Apoptosis in RBE cells (density plots). Data shown is average of three independent experiments. *P < 0.05.

To examine the effect of PEITC-CDDP co-treatment on cell viability, human gallbladder cancer GBC-SD cells were treated with PEITC, CDDP, or a PEITC-CDDP combination, and cell metabolic activity was measured using the 3-(4, 5-dimethylthia-zol-2-yl)-2, 5-diphenyl-tetrazolium bromide (MTT) assay. Notably, PEITC-CDDP co-treatment led to a significant reduction in cell viability (Figure 1A). To quantify synergism, the median-drug effect analysis method was used and the Combination Index (CI) values were calculated. Synergism is indicated by a CI of less than 1, additivity by a CI equal to 1, and antagonism by a CI greater than 1. Normalised isobolograms (Figure 1B) display data points below the additivity line, indicating synergy in growth inhibition of GBC-SD cells. Drug Reduction Index (DRI) was then calculated (Table 1). These results suggest that PEITC-CDDP co-treatment possesses a synergistic effect on GBC-SD cell proliferation.

**Table 1:** CI and DRI values of PEITC and CDDP in BTC cells

Cell line	PEITC (uM)	CDDP (ug/ml)	Fa^a^	CI^b^	DRI^c^ PEITC	DRI CDDP
GBC-SD	10	4	0.4282	0.661	6.157	2.006
RBE	8	8	0.2329	0.617	2.764	3.924

a Fa: fractional inhibition.

b CI < 1, *=* 1, and > 1 indicates synergism, additive effect, and antagonism, respectively.

c DRI: fold of dose reduction for each drug in combination, for a given degree of inhibition, when compared with the dose of each drug alone for the same degree of inhibition. A DRI greater than 1 indicates an enhanced cytotoxicity for the combination.

To determine whether PEITC in combination with CDDP decreases cell viability via an increase in apoptosis, annexin V-fluorescein isothiocyarate (Annexin V-FITC)/propidium iodide (PI) double labeling flow cytometry was used to determine the percentage of cells entering apoptosis. Flow cytometry analysis showed that CDDP caused about 10% of the cells to enter apoptosis, but co-treatment with PEITC dramatically enhanced CDDP-induced apoptosis to 40% (Figure 1C–1D). A similar pro-apoptotic effect of the combined treatment with PEITC and CDDP was also observed in human cholangiocarcinoma RBE cells (Figure 1E–1H). Together, these data demonstrated that PEITC can enhance CDDP-induced apoptosis in BTC cells.

### PEITC enhances the sensitivity of SP cells and xenograft tumors to CDDP

**Figure 2:**
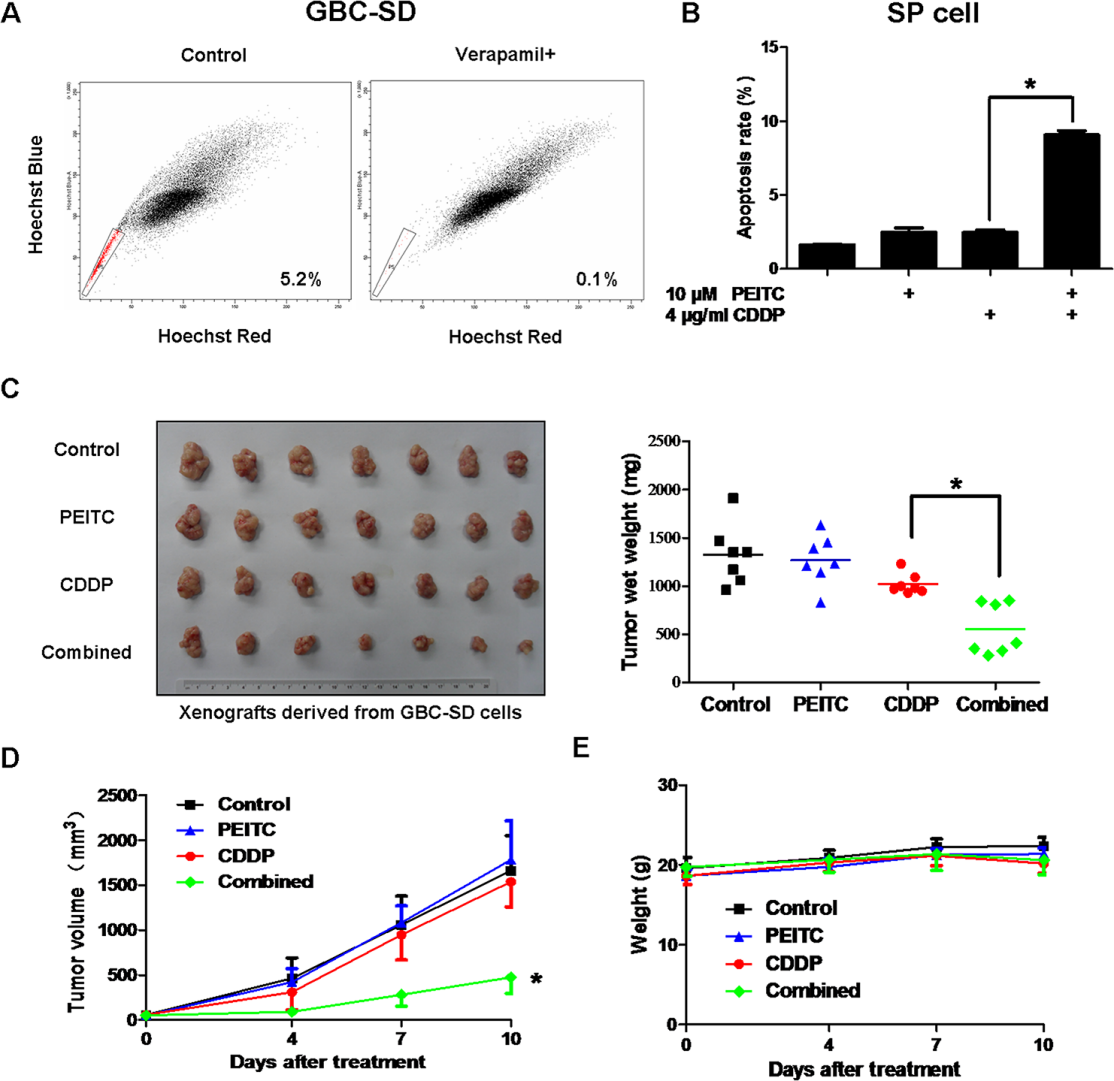
PEITC-CDDP co-treatment sensitizes SP cells and inhibits xenograft tumor growth without obvious toxic effects (**A**) FACS analysis on single cell suspension of GBC-SD cells stained with Hoechst 33342 dye showing SP cells. SP cells are enclosed within the area demarcated in black. Verapamil inhibited the efflux of the dye and caused the disappearance of SP cells. A representative plot of the frequency of SP cells is provided. (**B**) SP cells from GBC-SD cells were treated with PEITC, CDDP or PEITC-CDDP combination for 24 hrs and apoptosis detected by Annexin V/PI assay. Data shown is average of three independent experiments. *P < 0.05. (**C**) GBC-SD cells were transplanted into nude mice. When tumor size reached approximately 50 mm^3^, mice were randomly sorted into four equal groups. The tumor-bearing mice were intra-peritoneally injected with physiological saline as a control, PEITC, CDDP or PEITC-CDDP combination for 10 days. Xenografts were excised and weighed. Each dot represents weight of one tumor, and the mean tumor weights of each group is indicated by solid lines (right panel; *n =* 7). *P < 0.05. (**D**) Volume of the tumors was measured twice a week, and a tumor growth curve created for each group (*n =* 7). *P < 0.05,. (**E**) Mice were weighed twice a week, and a weight curve created for each group (*n =* 7).

Recent studies have shown that SP cells isolated from various cancer cell lines and primary tumors possess cancer stem-like properties [13–16]. SP cells can effectively avoid the effects of chemotherapeutic drugs, and are considered to be the root cause of tumor recurrence and metastasis. Therefore, we tested the effect of PEITC-CDDP co-treatment on SP cells from GBC-SD cells. The proportions of SP cells was 5.2% (Figure 2A). As shown in Figure 2B, flow cytometry analysis of SP cells treated with PEITC, CDDP, or a PEITC-CDDP combination showed that CDDP alone caused little cell apoptosis, but when combined with PEITC, markedly enhanced apoptosis at 24 hrs. These results demonstrate that PEITC significantly enhances the sensitivity of SP cells to CDDP.

To further examine the synergistic effect of PEITC and CDDP *in vivo*, GBC-SD cells were transplanted into nude mice. When the tumor size reached approximately 50 mm^3^, mice were randomly sorted into four equal groups. The tumor-bearing mice were intra-peritoneally injected with physiological saline as a control, PEITC, CDDP or PEITC-CDDP combination for 10 days. Treatment of mice with CDDP alone moderately inhibited tumor growth, but PEITC-CDDP combination treatment resulted in a striking reduction in the average tumor weight by about 50% (Figure 2C). The potent *in vivo* anticancer effect in the PEITC-CDDP combined group was further evident in the tumor growth curve data (Figure 2D). Systemic toxic effects of the treatments in these mice were evaluated by measuring the loss in body weight. No notable differences were observed between the treated groups (Figure 2E). Collectively, these results demonstrate that PEITC-CDDP co-treatment can effectively inhibit tumor growth without obvious toxic effects *in vivo*.

### Mcl-1 is partially responsible for CDDP resistance in GBC-SD and SP cells

**Figure 3:**
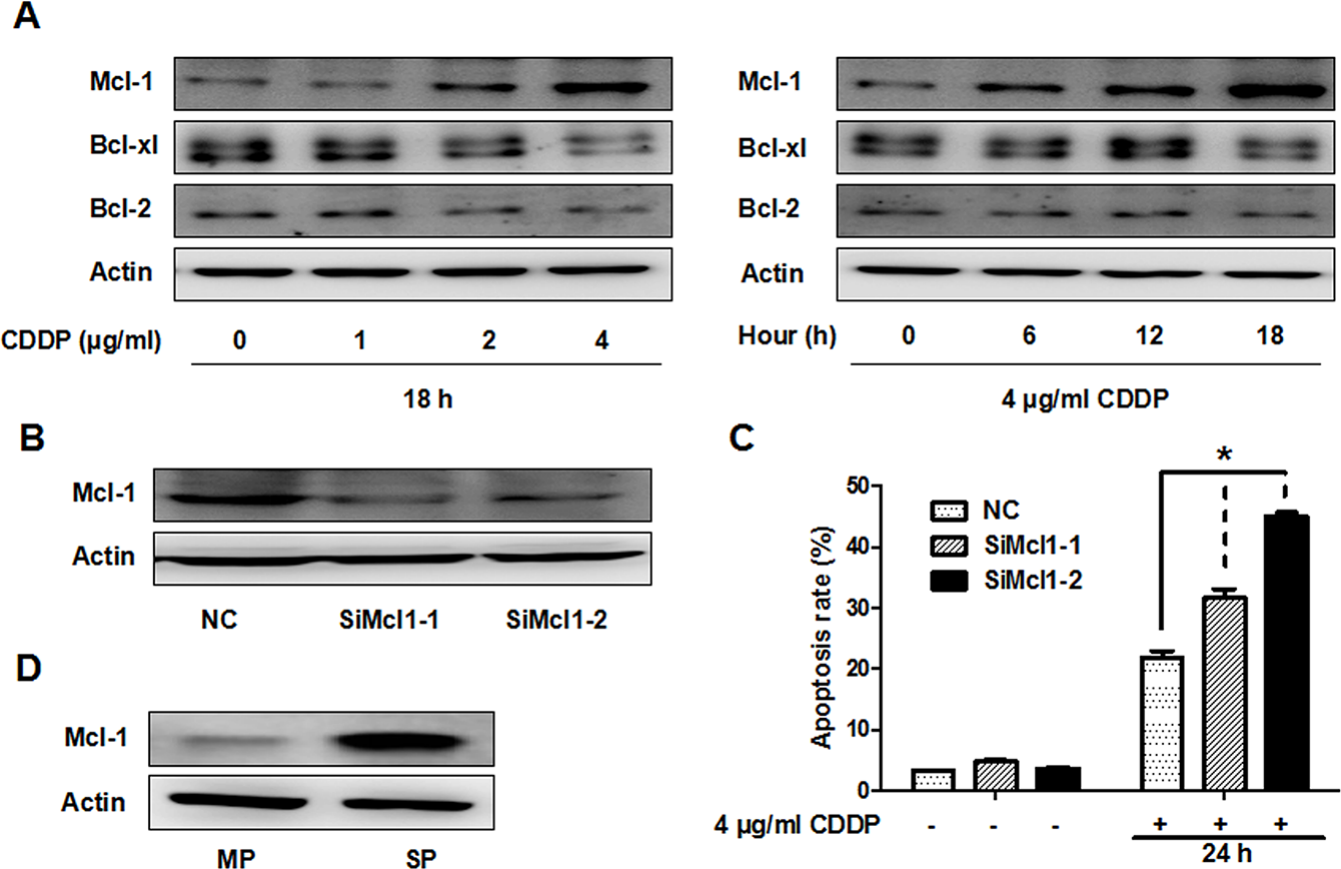
CDDP resistance is partially associated with Mcl-1 in GBC-SD and SP cells (**A**) Immunoblot analysis of Mcl-1, Bcl-xl and Bcl-2 protein in GBC-SD cells. Left panel, cells were treated with the indicated concentrations of CDDP for 18 hrs. Right panel, cells were treated with 4 ug/ml CDDP and harvested at the indicated times. Δ-Actin was used as a loading control. (**B**) Cells were transfected with non-specific siRNA (NC) or *Mcl-1* siRNA (SiMcl1) for 48 hrs and reduction in Mcl-1 was analysed by western blot (**C**) Apoptosis analysis using Annexin V/PI flow cytometry in GBC-SD cells transfected with *Mcl-1* siRNA after treatment with CDDP for 24 hrs. Data shown is average of three independent experiments. *P < 0.05. (**D**) Immunoblot analysis of Mcl-1 protein level in SP and MP cells from GBC-SD cells.

Anti-apoptotic proteins such as B-cell lymphoma 2 (Bcl-2), B-cell lymphoma-extra large (Bcl-xl) and Mcl-1 are known to play key roles in cancer cell apoptosis. Therefore, we investigated the relationship between these anti-apoptotic proteins and CDDP resistance in GBC-SD cells. Interestingly, CDDP treatment increased Mcl-1 protein level in a time- and dose-dependent manner, but did not increase Bcl-2 or Bcl-xl protein amounts (Figure 3A). To determine if Mcl-1 is playing a role in the cytotoxic sensitivity of GBC-SD cells to CDDP, cells were transfected with two siRNA oligonucleotides targeting Mcl-1 (Figure 3B), followed by CDDP treatment for 24 hrs. Knock down of Mcl-1 increased CDDP-induced apoptosis (Figure 3C). We also observed that Mcl-1 protein level was higher in SP cells than that in main population (MP) cells (Figure 3D). These data suggest that Mcl-1 is partially responsible for CDDP resistance in GBC-SD and SP cells.

### PEITC enhances the cytotoxicity of CDDP through proteasomal degradation of Mcl-1 *in vitro* and *in vivo*

**Figure 4:**
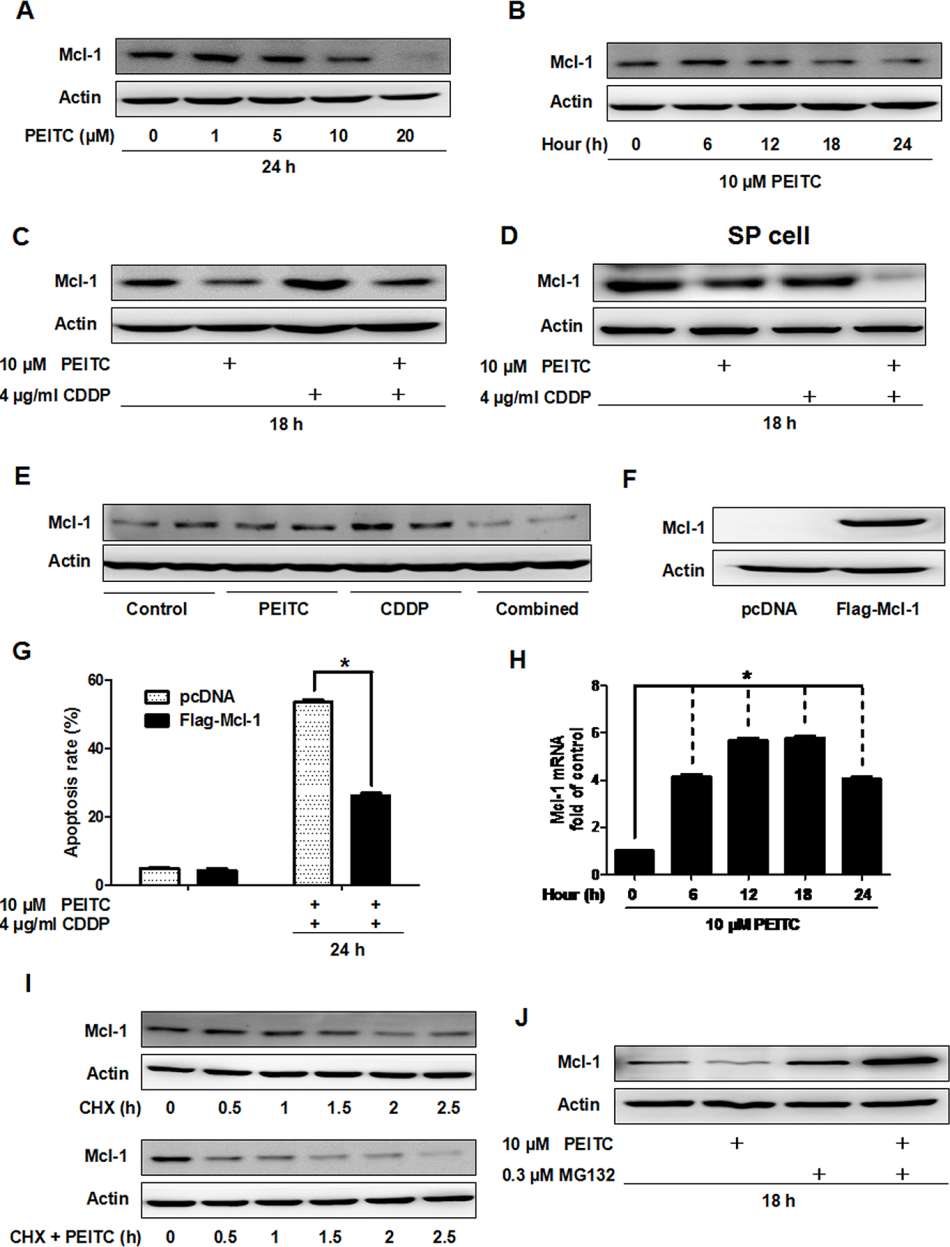
PEITC induces proteasomal degradation of Mcl-1 *in vitro* and *in vivo* (**A**) Immunoblot analysis of Mcl-1 in GBC-SD cells after treatment with the indicated concentrations of PEITC for 24 hrs. (**B**) Mcl-1 protein was analysed after treatement with 10 uM PEITC for various times (**C**) Mcl-1 protein level was analysed after treatment with PEITC, CDDP or PEITC-CDDP combination for 18 hrs. (**D**) SP cells from GBC-SD cells were treated with PEITC, CDDP or PEITC-CDDP combination for 18 hrs, and Mcl-1 protein level analyzed. (**E**) Immunoblot analysis of Mcl-1 protein in tumor tissue extracts from control group; PEITC group; CDDP group; and PEITC-CDDP combination group. Tumor tissues were from two mice of each group. (**F**) Mcl-1 protein level was determined by western blot after transfection with vector or *Mcl-1* plasmid for 48 hrs. (**G**) Apoptosis analysis using Annexin V/PI flow cytometry in GBC-SD cells transfected with *Mcl-1* plasmid after treatment with PEITC-CDDP combination for 24 hrs. (**H**) Time course analysis of *Mcl-1* mRNA by 10 uM PEITC in GBC-SD cells, detected by quantitative real time PCR analysis. Data shown is average of three independent experiments. *P < 0.05. (**I**) Immunoblot analysis of Mcl-1 protein in GBC-SD cells. Cells were treated with 20 ug/ml cycloheximide for the indicated times or were pretreated with 10 uM PEITC for 6 hrs before exposure to 20 ug/ml cycloheximide. (**J**) Mcl-1 protein was analysed in cells treated with PEITC, MG132 or PEITC-MG132 combination for 18 hrs. Δ-Actin was used as a loading control. CHX: cycloheximide.

To better understand the mechanism of how PEITC overcomes CDDP resistance, we analyzed Mcl-1 protein level in lysates from GBC-SD cells treated with PEITC and/or CDDP. PEITC treatment decreased Mcl-1 expression in a time- and dose-dependent manner (Figure 4A–4B). Notably, combined treatment with PEITC and CDDP significantly decreased Mcl-1 protein level compared to CDDP alone (Figure 4C). In SP cells, the PEITC-CDDP treatment also lead to a significant decrease in Mcl-1 protein level compared to treatment only with CDDP (Figure 4D). To investigate the effect of PEITC-CDDP treatment on Mcl-1 protein level *in vivo*, tumor tissues harvested from two mice of each group were examined by immunoblotting. PEITC and/or CDDP-mediated changes in Mcl-1 protein level in the tumor tissue were generally in agreement with the molecular alteration observed in cultured cells (Figure 4E). Furthermore, exogenous overexpression of Mcl-1 impeded PEITC-CDDP-induced apoptosis (Figure 4F–4G). These data suggest that PEITC enhances the cytotoxicity of CDDP through a reduction in Mcl-1 *in vitro* and *in vivo*.

To determine if the decrease in Mcl-1 protein level caused by PEITC was due to transcriptional inhibition or post-transcriptional regulation, we examined *Mcl-1* mRNA level by quantitative real time PCR in GBC-SD cells treated with PEITC. Surprisingly, PEITC increased *Mcl-1* mRNA level (Figure 4H). This suggested that PEITC-mediated decrease of Mcl-1 expression is regulated post-transcriptionally. Western blot analysis showed that Mcl-1 degradation was facilitated after 6 hours of PEITC treatment (Figure 4I). Next, to ask if PEITC mediated degradation of Mcl-1 involves proteasomal degradation, we treated GBC-SD cells with the proteasome inhibitor MG132 and found that the treatment recovered Mcl-1 protein amount to normal level (Figure 4J). Taken together, these data indicate that PEITC decreases Mcl-1 protein level via proteasomal degradation.

### PEITC induces proteasomal degradation of Mcl-1 through depletion of reduced glutathione (GSH) and decrease of GSH/oxidized glutathione (GSSG) ratio

**Figure 5:**
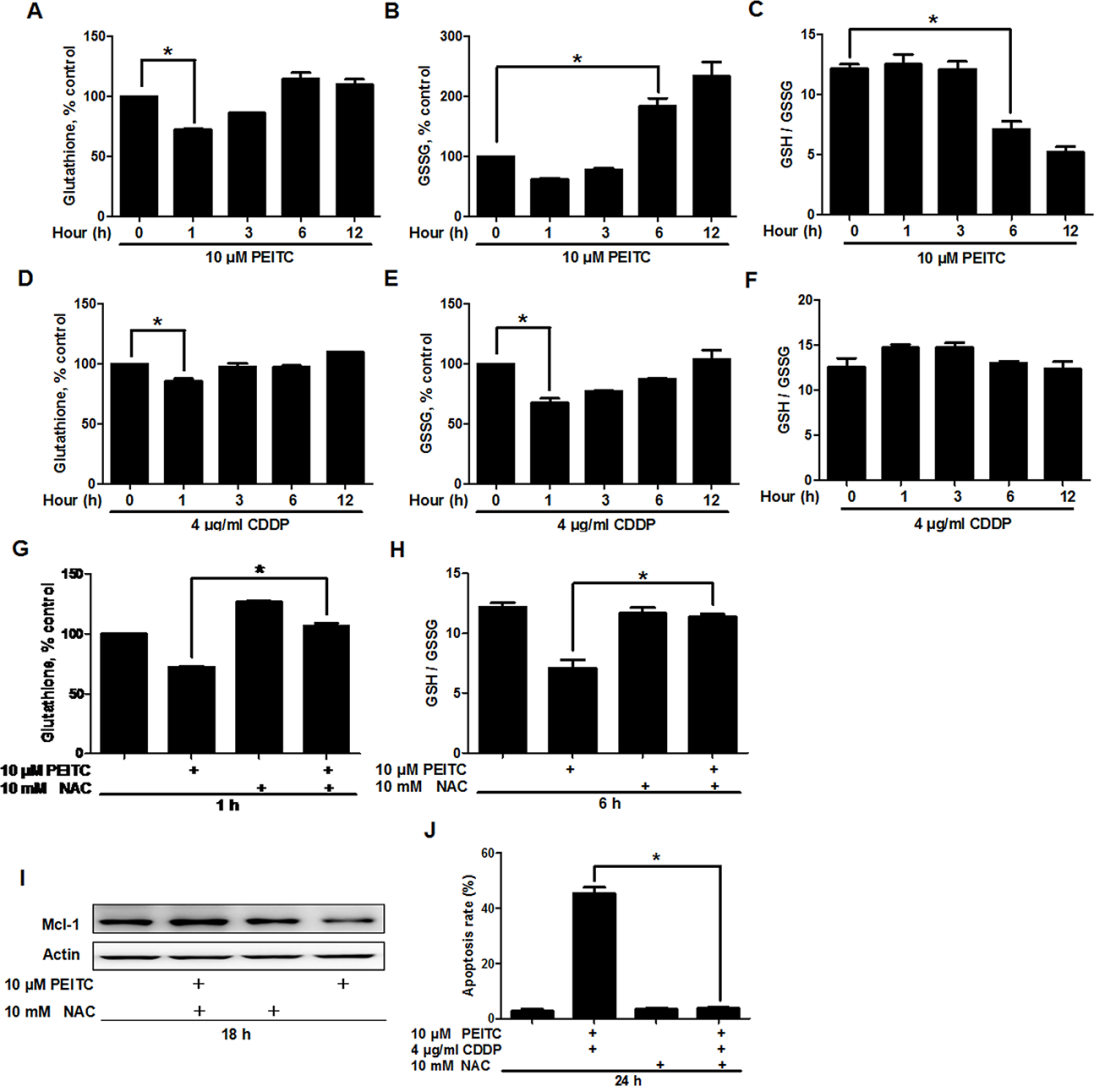
PEITC depletes GSH and decreases GSH/GSSG ratio Analysis of (**A**) GSH levels, (**B**) GSSG levels and (**C**) GSH/GSSG ratio in GBC-SD cells, after treatment with 10 uM PEITC for the indicated times. Analysis of (**D**) GSH levels, (**E**) GSSG levels and (**F**) GSH/GSSG ratio in GBC-SD cells treated with 4 ug/ml CDDP for the indicated times. (**G**) Effect of NAC on PEITC-induced GSH depletion. GBC-SD cells were preincubated with 10 mM NAC for 12 hrs before treatment with 10 uM PEITC for 1 hour. (**H**) Effect of NAC on PEITC-induced decrease in GSH/GSSG ratio. GBC-SD cells were preincubated with 10 mM NAC for 12 hrs before treatment with 10 uM PEITC for 6 hrs. (**I**) Effect of NAC on PEITC-induced Mcl-1 degradation. GBC-SD cells were preincubated with 10 mM NAC for 12 hrs before treatment with 10 uM PEITC for 18 hrs. Mcl-1 protein was detected by western blot and Δ-Actin was used as a loading control. (**J**) Effect of NAC on PEITC-CDDP-induced cell apoptosis. GBC-SD cells were preincubated with 10 mM NAC for 12 hrs before treatment with PEITC-CDDP combination for 24 hrs. Cell apoptosis was detected by Annexin V/PI assays. Data shown is average of three independent experiments. *P < 0.05.

Previous studies have shown that PEITC can alter the redox state of cancer cells through GSH reduction [6, 7, 17]. Since Mcl-1 is a redox sensitive protein [7], we analyzed the relationship between GSH reduction and Mcl-1 degradation in GBC-SD cells. Analysis of GSH revealed that PEITC induced a rapid GSH depletion, detectable after 1 hour of treatment (Figure 5A). As shown in Figure 5B–5C, PEITC increased GSSG levels and decreased GSH/GSSG ratio, which reflects the cellular redox state, after 6 hours of treatment. In comparison, CDDP only induced GSH and GSSG reduction with no apparent reduction in GSH/GSSG ratio (Figure 5D–5F). Therefore, these data suggest that PEITC can induce oxidative stress in GBC-SD cells. Coincidently, PEITC also facilitated Mcl-1 degradation after 6 hours of treatment (Figure 4B). Since there was no Mcl-1 degradation in the first few hours of PEITC incubation, it is likely that the depletion of GSH was a primary event that triggered a decrease in GSH/GSSG ratio and subsequent Mcl-1 degradation. In support of this hypothesis, supplementing cell culture medium with GSH precursor N-acetylcysteine (NAC) prevented PEITC-induced GSH depletion (Figure 5G), a decrease in GSH/GSSG ratio (Figure 5H), and Mcl-1 degradation (Figure 5I). Also, it significantly suppressed PEITC-CDDP-induced cell apoptosis (Figure 5J). Taken together, these data suggest that PEITC induces proteasomal degradation of Mcl-1 through depletion of GSH and a decrease in GSH/GSSG ratio.

### PEITC induces proteasomal degradation of Mcl-1 by increasing the glutathionylated Mcl-1

**Figure 6:**
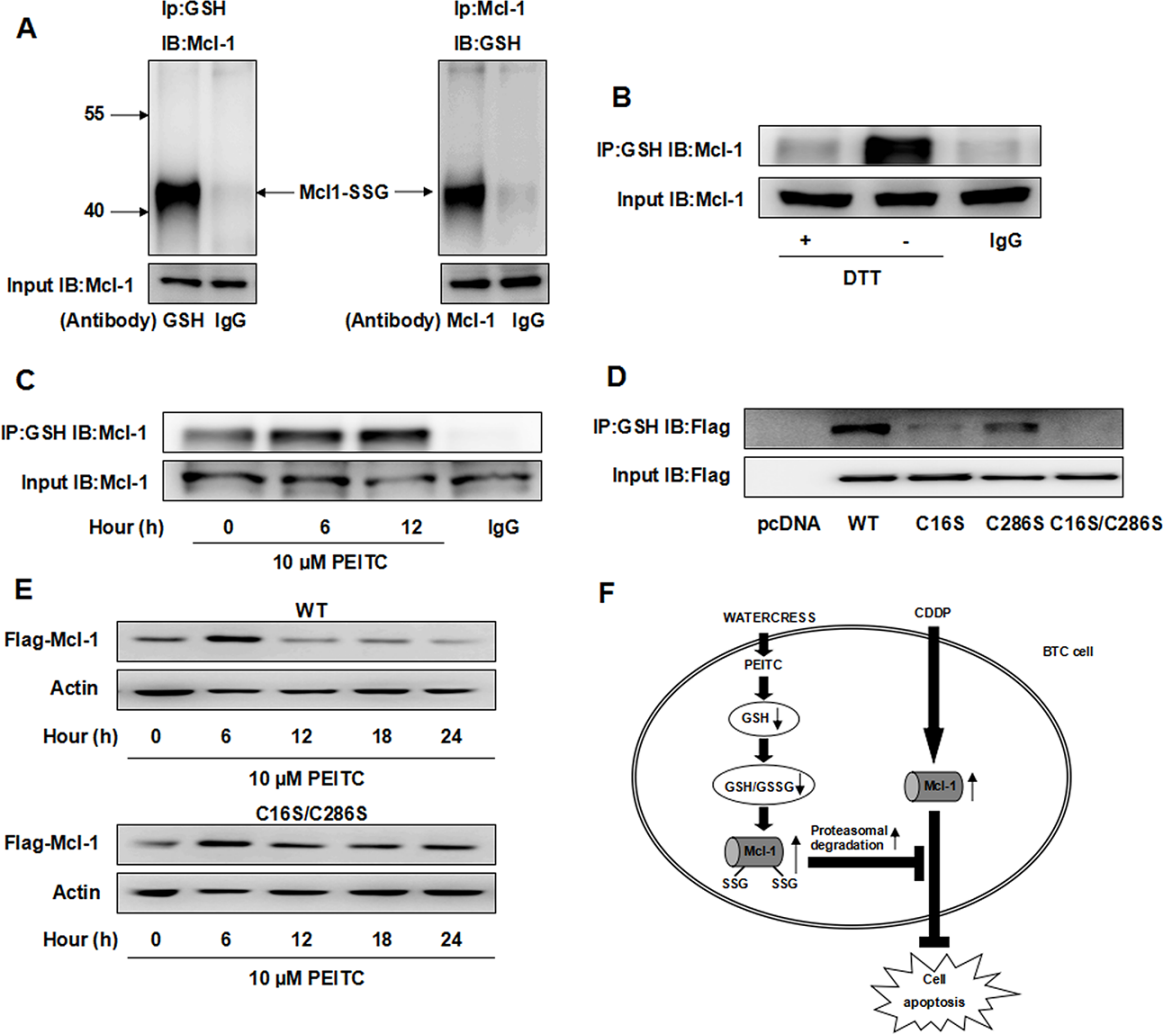
PEITC increases the glutathionylated Mcl-1 and induces glutathionylation-dependent degradation of Mcl-1 (**A**) Reciprocal IP/western analysis. Extracts from GBC-SD cells were immunoprecipitated using antibodies specific for Mcl-1 or GSH and the co-eluted proteins were detected by western blot with antibodies specific to GSH or Mcl-1. (**B**) GBC-SD cell lysate was treated with 10 mM DTT for 10 min before IP and the level of glutathionylated Mcl-1 was detected. (**C**) GBC-SD cells were treated with 10 uM PEITC and harvested at the indicated times and the level of glutathionylated Mcl-1 was detected by IP. (**D**) After transfection with Flag-Mcl-1 WT, C16S, C286S or C16S/C286S mutant for 48 hrs, level of glutathionylated Flag-Mcl-1 was detected by IP. (**E**) After transfection with Flag-Mcl-1 WT or C16S/C286S mutant for 48 hrs, GBC-SD cells were treated with 10 uM PEITC and harvested at the indicated times. Flag-Mcl-1 expression was detected by western blot and Δ-Actin was used as a loading control. (**F**) Proposed model of combination effect of PEITC and CDDP in BTC cells. CDDP can increase Mcl-1 protein level, which is partially responsible for CDDP resistance. PEITC can deplete GSH, decrease GSH/GSSG ratio and increase the glutathionylated Mcl-1, making Mcl-1 more susceptible to proteasomal degradation. Mcl1-SSG: the glutathionylated Mcl-1. IB: immunoblot.

Since Mcl-1 is a target of glutathionylation [7] and protein glutathionylation is greatly enhanced by decreased GSH/GSSG ratios that accompany cellular oxidative stress [18], we speculated that PEITC could increase the glutathionylated Mcl-1. Firstly, we found that endogenous Mcl-1 was partially glutathionylated under non-stressed conditions (Figure 6A). Furthermore, we found that DL-Dithiothreitol (DTT), a reducing agent, decreased the glutathionylated Mcl-1, and PEITC increased the glutathionylated Mcl-1 in a time-dependent manner (Figure 6B–6C).

Previous studies have demonstrated that glutathionylation of certain proteins may affect their functions and stability [18, 19]. We speculated that the glutathionylated Mcl-1 may be more susceptible to proteasomal degradation. Since only two cysteine residues, Cys16 and Cys 286, exist in the Mcl-1 protein, we investigated both these sites for potential glutathionylation. We used site-directed mutagenesis to convert these two cysteine residues Cys16 and Cys286, to serines (C16S, C286S, and C16S/C286S). By examining the glutathionylation of Flag-Mcl-1 wild type (WT) and mutants, we found that the C16S mutant was weakly glutathionylated and the C286S mutant was modestly glutathionylated (Figure 6D, lanes 3–4), whereas the double mutant was devoid of any glutathionylation (Figure 6D, lane 5). Taken together, these findings suggest that both the two cysteine residues of Mcl-1 are glutathionylation sites. Finally, cells expressing Flag-Mcl-1 WT and C16S/C286S mutant were treated with PEITC for 24 hrs, and their protein levels were determined. Flag-Mcl-1 WT protein was decreased in PEITC-treated cells, while the C16S/C286S mutant was not sensitive to PEITC-mediated degradation (Figure 6E), suggesting that PEITC induces glutathionylation-dependent degradation of Mcl-1.

## DISCUSSION

CDDP-based chemotherapy is an important treatment regimen used in the clinical management of BTC, but the mechanisms for CDDP resistance are not entirely clear. Cancer cells may become resistant to platinum-based drugs through multiple mechanisms, such as an increased ability to repair DNA damage caused by platinum, neutralization of platinum toxicity, blocking platinum entry into the nucleus, an increase in drug export and so on [11, 20–24]. In addition, cisplatin resistance can develop through an increased ability to avoid drug-induced cell damage, cell shrinkage and hence initiation of apoptosis [25]. Apoptosis is regulated in part by the Bcl-2 family of proteins which consists of both pro-apoptotic and anti-apoptotic proteins [26]. Among all the anti-apoptotic Bcl-2 family members, Mcl-1 functions as a major survival factor, particularly in solid cancers [27]. Despite the confirmed importance of Mcl-1 in several cancers, the role of Mcl-1 in BTC survival has yet to be explored. In this study, we provided evidence that the effectiveness of CDDP is partially dependent on the Mcl-1 expression. Previously we found that Mcl-1 expression is increased in gallbladder carcinoma tissues [28]. Therefore investigation into new therapeutic strategies targeting Mcl-1 could prove crucial in treating BTC.

Several studies have discovered that PEITC is able to enhance the cytotoxic effect of CDDP in cancer cells [10, 11]. But the synergistic effect of PEITC and CDDP in BTC cells remains unknown. Moreover, little information is available concerning the functional role of Mcl-1 in mediating PEITC-induced chemosensitization. In this study, we found that PEITC can significantly enhance CDDP-induced apoptosis by degrading Mcl-1 in BTC cells. Under mild oxidative stress, protein glutathionylation regulates functions of multiple proteins [18, 19]. Here, our results clearly show that Mcl-1 is glutathionylated in GBC-SD cells and a sub-toxic concentration of PEITC increases the glutathionylated Mcl-1, followed by rapid proteasomal degradation. Whereas, Trachootham et al. showed that PEITC renders Mcl-1 more susceptible to cleavage by caspase-3 by de-glutathionylation of Mcl-1 in chronic lymphocytic leukemia cells [7]. We speculate that the discrepancy is due to different cell lines and different concentration of PEITC in two studies. PEITC may have different effect on pathways existing in different cells, and may impact degradation pathway of Mcl-1. By mutagenesis, we identified and confirmed that both Cys16 and Cys286 are glutathionylation sites on human Mcl-1 protein. Although there was a consistent correlation between glutathionylation levels and proteasomal degradation of Mcl-1, the double mutant could not be glutathionylated (Figure 6D) and only partially prevented proteasomal degradation (Figure 6E). This suggests that PEITC-mediated degradation of Mcl-1 may involve not only glutathionylation-dependent degradation but also other mechanisms. It would be interesting to examine other mechanisms of PEITC-mediated degradation of Mcl-1.

Conventional chemotherapies for cancer cells are believed to mainly eliminate the majority of differentiated cancer cells but spare cancer stem cells, which are thought to be associated with recurrence [29–31]. In the present study, we used SP cells from GBC-SD cells as a model of cancer stem-like cells and found that SP cells have higher Mcl-1 expression, consistent with a recent report [32]. Moreover, PEITC enhanced the efficacy of CDDP by degrading Mcl-1 in SP cells, which implies that PETIC is a promising chemotherapy-sensitizing agent targeting cancer stem cells.

Previous studies showed that eating watercress can significantly increase the blood level of PEITC in humans [9] and an oral dose of 40 mg PEITC can result in a plasma concentration in the micromolar range within 3 to 8 hrs [33]. Thus, the effective concentration of PEITC appears to be achievable by oral supplementation in humans. Furthermore, a Phase I trial showed that at lower doses (40 and 80 mg daily for 30 days) PEITC was well-tolerated, and patients that consumed high doses of PEITC (120 and 160 mg daily for 30 days) showed only minor toxicity with low-grade diarrhea [5]. The fact that PEITC is found in our normal diets combined with its relatively low toxicity in humans provides strong support for a clinical investigation of PEITC-CDDP co-treatment in BTC.

In conclusion, CDDP resistance is partially associated with Mcl-1 in BTC cells and PEITC can enhance CDDP-induced apoptosis via glutathionylation-dependent degradation of Mcl-1. These results not only identify a novel mechanism of PEITC-enhanced chemosensitivity of BTC cells to CDDP, but also provide support that dietary intake of watercress may help reverse CDDP resistance in BTC patients.

## MATERIALS AND METHODS

### Cell culture and reagents

The human gallbladder cancer (GBC-SD) and human cholangiocarcinoma (RBE) cell lines were obtained from Cell Bank, Shanghai Institutes for Biological Sciences, Chinese Academy of Sciences. GBC-SD and RBE cells were maintained in RPMI 1640 (Hyclone) supplemented with 10% fetal bovine serum (Hyclone). Cells were cultured in a humidified atmosphere of 5% CO_2_ at 37°C.

PEITC, NAC, Hoechst 33342, verapamil, DTT and cycloheximide were purchased from Sigma. MG132 was purchased from Calbiochem and CDDP was obtained from Qilu Pharmaceutical Co., Ltd (Jinan, China). PEITC was dissolved in dimethyl sulfoxide (DMSO) and was freshly diluted in culture media before use in experiments.

### SP cell sorting from GBC-SD cells

SP analysis was performed as previously described [34]. Briefly, cells were trypsinized and resuspended in ice-cold Hank's balanced salt solution (Invitrogen). Hoechst 33342 was added at a final concentration of 5 mg/ml in the presence or absence of 50 mg/ml verapami. After incubating at 37°C for 90 min, the cells were analyzed by flow cytometry.

### Cell viability, apoptosis analysis, CI and DRI

Cell viability was assayed using the MTT assay (Sigma) as previously described [35]. Cell apoptosis was assessed using an Annexin V-FITC/PI kit (BD Pharmingen) and analyzed by flow cytometry on FACS Calibur (Becton Dickson) [36]. Cells that were positively stained by Annexin V-FITC only (early apoptosis) and positive for both Annexin V-FITC and PI (late apoptosis) were quantitated and both subpopulations were considered as overall apoptotic cells.

CI and DRI values of PEITC-CDDP co-treatment were determined using the CalcuSyn software using non-constant ratio combination design as previously described [37, 38].

### Reverse transcription and real-time PCR

Reverse transcription and quantitative real-time PCR were carried out as previously described [39]. Primers sequences used in PCR analysis were as follows: *Mcl-1* forward 5′-TCCAAGGCATGCTTCGGA-3′ and reverse 5′-GGCACCAAAAGAAATGAGAGTCAC-3′; *GAPDH* forward 5′-GAAGGTGAAGGTCGGAGTC-3′ and reverse 5′-GAAGATGGTGATGGGATTTC-3′. Human *GADPH* mRNA served as an endogenous control for normalization.

### GSH/GSSG ratio assay

GSH is a tripeptide with a free thiol group and function as a major antioxidant in cells. GSH/GSSG ratio reflects the cellular redox state. The total GSH and GSSG were determined by colorimetric microplate assay kit (Beyotime, China) as previously described [40, 41]. GSH/GSSG ratio was obtained {ratio = (Total GSH – 2GSSG)/GSSG}.

### *Mcl-1* siRNA and mutant transfection

Two specific siRNA oligonucleotides complementary to the *Mcl-1* mRNA sequence were transiently transfected into GBC-SD cells using Lipofectamine 2000 (Invitrogen) as previously described [42, 43]. A non-specific siRNA was also transfected as a mock control. After 48 hrs, GBC-SD cells were either lysed for western blot analysis or were exposed to CDDP for an additional 24 hrs and analysed for apoptosis using the methods described above. The siRNA-1 sequences complimentary for *Mc1–1* mRNA were 5′ GUAUCACAGACGUUCUCGUdTdT 3′ and 3′ dTdTCAU AGUGUCUGCAAGAGCA 5′. The siRNA-2 sequences complimentary for *Mcl-1* mRNA were 5′ GGACUUUU AGAUUUAGUGAdTdT 3′ and 3′ dTdTCCUGAAAAUC UAAAUCACU 5′.

Site-directed mutagenesis of the *Mcl-1* gene was performed using the Quick Change Lightning Site-Directed Mutagenesis Kit (Agilent Technologies) using the pcDNA3.1-Flag-Mcl-1 plasmid as the template. Cells were transiently transfected with Flag-Mcl-1 WT and mutants using the above-mentioned Lipofectamine method. Nonrelevant plasmid pcDNA3.1 was used as the transfection control. After 48 hrs, GBC-SD cells were lysed for immunoprecipitation (IP) or treated with PEITC for an additional 24 hrs and analysed by western blot. The anti-Flag monoclonal antibody was purchased from Sigma.

### Assays of Mcl-1 expression and glutathionylation

Mcl-1, Bcl-xl and Bcl-2 protein levels were determined by western blot as previously described [44]. Cellular glutathionylation of Mcl-1 was determined by IP with an anti-GSH antibody under non-reducing conditions, followed by western blot analysis [7, 18] using an anti-Mcl-1 antibody. Antibodies to Bcl-2 and Bcl-xl were purchased from Santa Cruz. Antibodies to GSH and Mcl-1 were purchased from Abcam.

### *In vivo* study in tumor-bearing mice

All animal experiments were done in accordance with institutional guidelines for animal welfare. GBC-SD cells were harvested, washed, and resuspended in serum-free RPMI 1640 and then injected subcutaneously into 6-week old BALB/c-nu/nu mice (*n =* 28 mice, purchased from Shanghai Experimental Animal Center). Tumor volumes were measured twice a week using a calliper, and were calculated using the formula V *=* π/6 × length × width^2^ [34, 45]. When the tumor size was approximately 50 mm^3^, the mice were sorted into four equal groups (*n =* 7 mice per group). The tumor-bearing mice were intra-peritoneally administered with physiological saline as a control, PEITC (25 mg/kg), CDDP (2.5 mg/kg) or PEITC/CDDP twice a week. Tumor volumes were measured twice a week with a calliper and body weights were also recorded. The mice were sacrificed after 10 days, and body weight and tumor weight were measured.

### Statistical analysis

Data were shown as mean value ± SD. SPSS17.0 software was used for statistical analysis. Analysis of variance was applied for comparison of the means of two or multiple groups. A value of *P* < 0.05 was considered significant.
